# Blue Light-Initiated Alcoholic RAFT Dispersion Polymerization of Benzyl Methacrylate: A Detailed Study

**DOI:** 10.3390/polym11081284

**Published:** 2019-08-01

**Authors:** Dongdong Liu, Ruiming Zeng, Hao Sun, Li Zhang, Jianbo Tan

**Affiliations:** 1 Department of Polymeric Materials and Engineering, School of Materials and Energy, Guangdong University of Technology, Guangzhou 510006, China; 2 Department of Chemistry, Northwestern University, Evanston, IL 60208, USA; 3 Guangdong Provincial Key Laboratory of Functional Soft Condensed Matter, Guangzhou 510006, China

**Keywords:** alcoholic RAFT dispersion polymerization, blue light, diblock copolymer nano-objects

## Abstract

Blue light-initiated alcoholic reversible addition-fragmentation chain transfer (RAFT) dispersion polymerization of benzyl methacrylate (BzMA) using bis (acyl) phosphane oxide (BAPO) as the photo-initiator is developed to prepare diblock copolymer nano-objects. High monomer conversion (95%) was achieved within 2 h of blue light irradiation in an isopropanol/water mixture. Effects of solvent, light intensity, and reaction temperature on the polymerization kinetics were evaluated. Finally, the effect of reaction temperature on the morphologies of diblock copolymer nano-objects was investigated and two morphological phase diagrams were constructed at 25 and 70 °C. Transmission electron microscopy (TEM) measurement confirmed that increasing the reaction temperature promoted the evolution of higher order morphology. We believe this study will provide more mechanistic insights into alcoholic RAFT dispersion polymerization for the creation of diblock copolymer nano-objects with well-defined structures.

## 1. Introduction

Solution self-assembly of block copolymers is a versatile method to produce block copolymer nano-objects with different morphologies, including spheres, cylindrical micelles, vesicles, lamellae etc., [1,2,3]. These block copolymer nano-objects have diverse applications in nanoreactors, catalysis, bioimaging, and drug delivery [4,5,6,7]. However, the solution self-assembly of block copolymers is usually conducted at diluted block copolymer concentrations (<1%). Moreover, additional post-polymerization processing (pH change, solvent switch) is usually required [1]. These features restrict the production of block copolymer nano-objects in a large scale by solution self-assembly, which is not beneficial for industrial applications.

In recent years, polymerization-induced self-assembly (PISA) via reversible addition-fragmentation chain transfer (RAFT) dispersion polymerization has become a robust method for the production of block copolymer nano-objects with various morphologies at high solids (10–50%) [8,9,10,11,12,13]. In the case of RAFT dispersion polymerization, the monomers should be fully dissolved in the reaction medium while the generated polymers are insoluble. Block copolymer nano-objects with diverse morphologies have been produced in a variety of solvents including water [9,14,15,16], alcoholic solvents (alcohols or alcohol/water mixtures) [17,18,19], poly(ethylene glycol) [20,21], non-polar solvents [22,23,24], ionic liquid [25], and supercritical carbon dioxide [26]. In particular, alcoholic RAFT dispersion polymerization has become one of the most commonly studied PISA techniques to produce well-defined block copolymer nano-objects due to its many advantages. First, the library of monomers that are suitable for alcoholic RAFT dispersion polymerization is particularly large, allowing for the preparation of a variety of block copolymer nano-objects [27]. Second, the problem of electrostatic repulsion between highly charged stabilizers in aqueous self-assembly can be solved in alcoholic RAFT dispersion polymerization, facilitating the creation of charged block copolymer nano-objects with higher order morphologies. Third, lower surface tension of alcohol is beneficial for the deposition of polymer particles on planar substrates [18]. 

Several research groups have extensively studied alcoholic RAFT dispersion polymerization over the past several years. Pan and co-workers [17] reported the first alcoholic RAFT dispersion polymerization of styrene at 80 °C that can generate higher order structures. However, incomplete monomer conversions (70% or lower) were observed even within 48 h. This can be explained by the fact that the reactivity of styrene is relatively low. As an alternative, the Armes Group [18] reported the alcoholic RAFT dispersion polymerization of benzyl methacrylate (BzMA) in ethanol at 70 °C and high monomer conversion (>98%) was observed within 24 h. Since then, BzMA has become one of the most popular monomers in alcoholic RAFT dispersion polymerization [27]. However, most alcoholic RAFT dispersion polymerizations of BzMA were conducted at high temperatures using a thermal initiator. This feature of alcoholic RAFT dispersion polymerization restricts the incorporation of thermally-sensitive species into block copolymer nano-objects and the development of new PISA formulations [28,29]. Fortunately, the recent development of photoinitiated polymerization-induced self-assembly (photo-PISA) via visible light-initiated RAFT dispersion polymerization developed by our group and others [19,30,31,32,33,34,35,36,37,38,39,40,41,42,43,44,45] has allowed the production of block copolymer nano-objects at low temperatures. The Boyer group [36] reported the first visible light-induced RAFT dispersion polymerization of BzMA in alcoholic solvents at room temperature. However, the polymerization rate is relatively low, e.g., low monomer conversions (40–70%) were achieved within 24 h [36]. Moreover, acetonitrile (MeCN), a toxic cosolvent was usually required to prepare higher order morphology [37,39].

In this contribution, blue light-initiated alcoholic RAFT dispersion polymerization of BzMA was developed to prepare well-defined diblock copolymer nano-objects at room temperature. BAPO, a commercially available photo-initiator, was employed in this case. Effects of reaction parameters including solvent, light intensity, and reaction temperature on alcoholic RAFT dispersion polymerization of BzMA were studied in detail. This study will provide more mechanistic insights into alcoholic RAFT dispersion polymerization for the production of block copolymer nano-objects.

## 2. Results and Discussion

### 2.1. Synthesis of Macro-CTAs

In this study, two macromolecular chain transfer agents (macro-CTAs) were selected to mediate the blue light-initiated alcoholic RAFT dispersion polymerization of BzMA. Monomethoxy poly (ethylene glycol) (mPEG)-based macro-CTA has been employed in aqueous or alcoholic RAFT dispersion polymerization due to its excellent solubility in both aqueous and alcoholic solvents [19,31,46]. Herein, mPEG-based macro-CTA was prepared by esterification of mPEG_45_ and 4-cyano-4-(dodecylsulfanylthiocarbonyl) sulfanylpentanoic acid (CDPA), denoted as mPEG_45_-CDPA. An esterification efficiency of 96% was confirmed by ^1^H NMR spectroscopy (Figure S1). Poly (hydroxypropyl methacrylate)-CTA (PHPMA-CTA) was prepared by RAFT solution polymerization of HPMA in 1,4-dioxane at 70 °C using azobisisobutyronitrile (AIBN) as the initiator and CDPA as the CTA. The polymerization was quenched after 2 h (monomer conversion = 90% as measured by ^1^H NMR measurement) and a mean degree of polymerization (DP) of 18 was confirmed (denoted as PHPMA_18_-CDPA). Tetrahydrofuran (THF) gel permeation chromatography (GPC) gave *M*_n_ = 4.2 kg/mol and *M*_w_/*M*_n_ = 1.15.

### 2.2. Blue Light-Initiated Alcoholic RAFT Dispersion Polymerization of BzMA

**Scheme 1:**
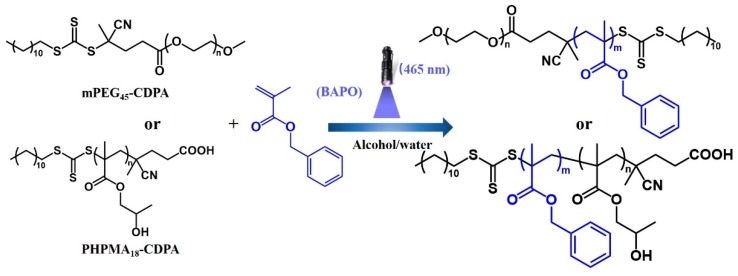
Preparation of PBzMA-based diblock copolymer nano-objects via blue light-initiated alcoholic reversible addition-fragmentation chain transfer (RAFT) dispersion polymerization of BzMA with mPEG_45_-CDPA or PHPMA_18_-CDPA as the macro-chain transfer agents (CTA) and BAPO as the photo-initiator.

The macro-CTAs (mPEG_45_-CDPA and PHPMA_18_-CDPA) were then utilized to chain extend with BzMA to produce a variety of diblock copolymer nano-objects via blue light-initiated RAFT dispersion polymerization in alcohol/water mixtures (Scheme 1). BAPO, a commercially available photo-initiator, was employed to initiate the polymerization. In this study, the molar ratio of macro-CTA and BAPO was maintained at 3.0 to ensure high polymerization rate and good controllability.

**Figure 1:**
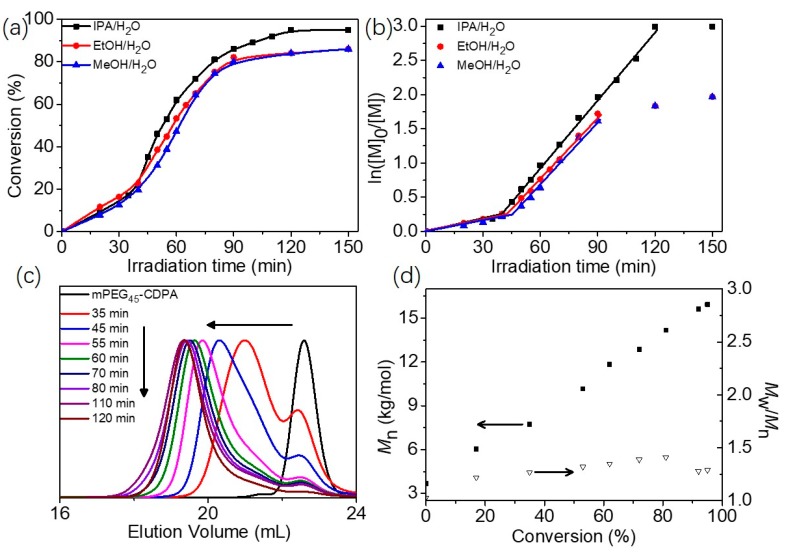
(**a**) Polymerization kinetics for blue light-initiated alcoholic RAFT dispersion polymerization of mPEG_45_-PBzMA_100_ with 20% *w*/*w* BzMA in different solvents. (**b**) Plots of ln ([M]_0_/[M]) versus irradiation time according to the data in Figure 1a. (**c**) THF GPC traces of mPEG_45_-CDPA and diblock copolymers prepared via blue light-initiated alcoholic RAFT dispersion polymerization of mPEG_45_-PBzMA_100_ with 20% *w*/*w* BzMA in isopropanol/water (80/20, *w*/*w*). (**d**) Evolution of *M*_n_ and *M*_w_/*M*_n_ with monomer conversion for the blue light-initiated alcoholic RAFT dispersion polymerization of mPEG_45_-PBzMA_100_ with 20% *w*/*w* BzMA in isopropanol/water (80/20, *w*/*w*). All experiments were carried out at room temperature.

Blue light-initiated alcoholic RAFT dispersion polymerization of BzMA was first conducted in an ethanol/water mixture (80/20, *w*/*w*) at room temperature using mPEG_45_-CDPA as the macro-CTA (*λ*_max_ = 465 nm, 4.2 mW/cm^2^). The transparent reaction mixture became bluish and the subsequent milky white as the polymerization proceeded, indicating the in situ formation of polymer nanoparticles. However, the final monomer conversion was relatively low (<90%) within 2 h of blue light irradiation. In RAFT dispersion polymerization, the monomers should be fully dissolved in the reaction medium. Therefore, the solvent used in RAFT dispersion polymerization should have a significant impact on the PISA process as well as the final morphology [47,48]. Kinetic studies of blue light-initiated alcoholic RAFT dispersion polymerizations of BzMA (20% *w*/*w*) were then conducted in different alcohol/water mixtures (80/20, *w*/*w*) using mPEG_45_-CDPA as the macro-CTA (target DP of 100), as shown in Figure 1a. It is found that the blue light-initiated RAFT dispersion polymerization of BzMA conducted in isopropanol/water was significantly faster than that in ethanol/water or methanol/water. Moreover, the final monomer conversion reached up to 95% in isopropanol/water while the final monomer conversions were only 86% in both ethanol/water and methanol/water. According to the Hildebrand solubility parameters, isopropanol (δ = 23.8 [MPa]^1/2^) should be a relatively better solvent for the growing PBzMA chains (δ = 15.3 [MPa]^1/2^) than that of ethanol (δ = 26.2 [MPa]^1/2^) or methanol (δ = 29.7 [MPa]^1/2^) [48]. Therefore, the PBzMA chains in isopropanol/water should have higher mobility and be easier to be swollen, leading to the relatively high monomer concentration in the particles and therefore the observed faster polymerization behavior even at high monomer conversions. The change of alcoholic solvent should have little effect on the polymerization rate in the homogenous polymerization stage, since the formed mPEG_45_-PBzMA diblock copolymers are fully dissolved in the solvent. This can be further confirmed by the semilogarithmic plots. Two distinct regimes were observed in all cases (Figure 1b), which correspond to the homogenous polymerization and heterogenous polymerization. The polymerization rate was almost identical in each case during the homogenous polymerization stage. During the heterogenous polymerization stage, the polymerization mainly occurred inside the monomer-swollen micelles, leading to the increase in polymerization rate. In the following experiments, most polymerizations were conducted in isopropanol/water (80/20, *w*/*w*) unless otherwise stated.

Samples withdrawn during the kinetic study of blue light-initiated RAFT dispersion polymerization in isopropanol/water were also characterized by THF GPC. Figure 1c shows corresponding GPC traces of the samples and two GPC peaks were found in the GPC trace at the beginning of the polymerization (e.g., 35 min). The right peak should correspond to the unreacted mPEG_45_-CDPA, while the left peak should correspond to the formed mPEG_45_-PBzMA_n_ diblock copolymer. As the polymerization proceeded, the left peak decreases gradually and the right peak shifts to lower elution volume. Near unimodal GPC trace was observed for the final sample although a small amount of mPEG_45_-CDPA still existed. The evolution of number-average molecular weight (*M*_n_) with monomer conversion was linear. Moreover, narrow molecular weight distributions (*M*_w_/*M*_n_ < 1.41) were confirmed as the polymerization proceeded. A *M*_w_/*M*_n_ value of 1.29 was observed for the final sample (Figure 1d). The non-zero y-intercept is attributed to the mPEG_45_-CDPA, which has a *M*_n_ value of 3.7 kg/mol as measured by THF GPC. These results suggest that good control was maintained in the blue light-initiated alcoholic RAFT dispersion polymerization when using BAPO as the photo-initiator.

**Figure 2:**
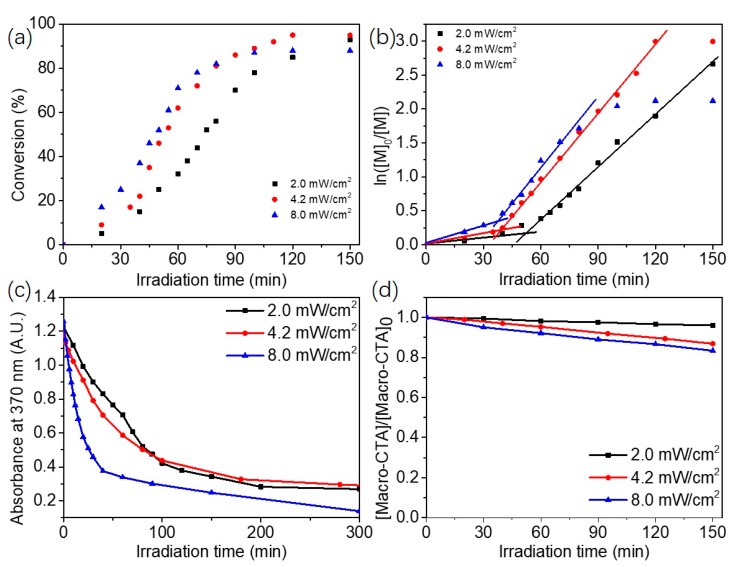
(**a**) Polymerization kinetics for blue light-initiated alcoholic RAFT dispersion polymerization of mPEG_45_-PBzMA_100_ (20% *w*/*w* BzMA) at different light intensities. (**b**) Plots of ln ([M]_0_/[M]) versus irradiation time for blue light-initiated alcoholic RAFT dispersion polymerization of mPEG_45_-PBzMA_100_ (20% *w*/*w* BzMA) at different light intensities. (**c**) Plots of normalized absorbance (at 370 nm) of BAPO vs. irradiation time under 465 nm visible-light irradiation. (**d**) Degradation of mPEG_45_-CDPA under blue light irradiation as measured by UV–Vis spectroscopy at different light intensities.

We then explored the effect of light intensity on the blue light-initiated alcoholic RAFT dispersion polymerization of BzMA. Figure 2a shows the effect of light intensity on polymerization kinetics of blue light-initiated alcoholic RAFT dispersion polymerization of BzMA (target DP of 100, 20% *w*/*w* BzMA). It is obvious that higher light intensity led to faster polymerization. This was also confirmed by the corresponding plots of ln ([M]_0_/[M]) vs. irradiation time (Figure 2b). The evolution of UV–Vis absorbance of BAPO at 370 nm with blue light irradiation time was also checked (Figure 2c), and faster decomposition of BAPO was observed at higher light density. Therefore, the higher rate of polymerization can be explained by the faster production of free radicals at higher light intensity. It should be noteworthy that the final monomer conversion was relatively low (88%) for the strongest blue light irradiation (8.0 mW/cm^2^). This may be attributed to the fast production of radicals under 8.0 mW/cm^2^, leading to the increased probability of biradical termination. Moreover, the degradation of mPEG_45_-CDPA was also checked by UV–Vis spectroscopy (Figure 2d). It is shown that higher blue light intensity led to faster degradation of mPEG_45_-CDPA, and this should also contribute to the polymerization kinetics of blue light-initiated alcoholic RAFT dispersion polymerization. THF GPC measurement confirmed that the change of light intensity had little effect on the molecular weight and molecular weight distribution on the obtained diblock copolymers (Table S1). The present method provides a facile platform to control the polymerization rate by just changing the light intensity, which is difficult for traditional thermally initiated RAFT dispersion polymerization.

Typically, alcoholic RAFT dispersion polymerization is conducted at high temperature (e.g., 70 °C) via the decomposition of thermal initiators [17,18,48]. This feature hinders the production of polymer nano-objects that contain temperature-sensitive species (e.g., epoxy group, antibody). Moreover, the decomposition of thermal initiator is very sensitive to the change of temperature, which makes the study of effect of temperature on PISA challenging. One of the main advantages of visible light-initiated RAFT dispersion polymerization is that high monomer conversions can be achieved at different temperatures within a short irradiation time [49]. Therefore, the visible light-initiated RAFT dispersion polymerization provides an opportunity to investigate the effect of temperature on PISA, including polymerization kinetics and morphologies. Herein, the sole effect of temperature on blue light-initiated alcoholic RAFT dispersion polymerization of BzMA was also investigated.

**Figure 3:**
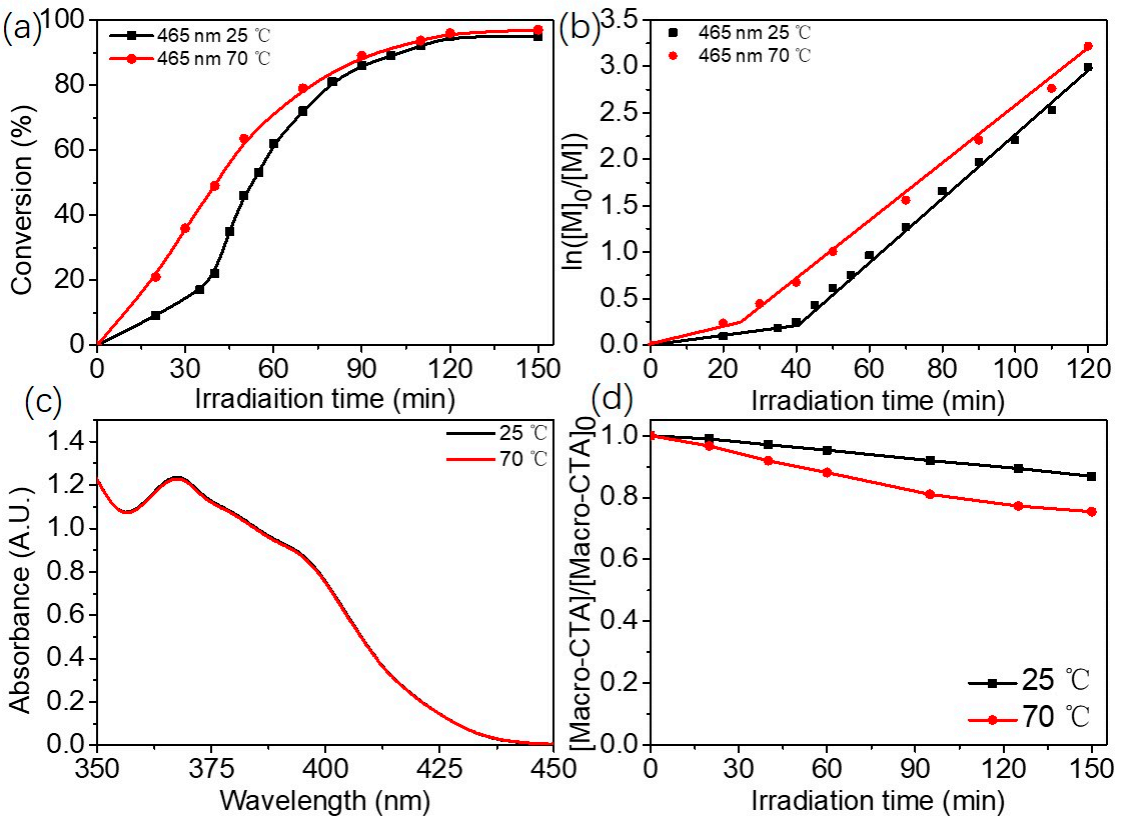
(**a**) Polymerization kinetics of blue light-initiated RAFT dispersion polymerization of mPEG_45_-PBzMA_100_ in isopropanol/water (80/20, *w*/*w*) at different temperatures (20% *w*/*w* BzMA). (**b**) Plots of ln ([M]_0_/[M]) versus irradiation time for blue light-initiated RAFT dispersion polymerization of mPEG_45_-PBzMA_100_ in isopropanol/water (80/20, *w*/*w*) at different temperatures (20% BzMA). (**c**) UV–Vis absorbance spectra of BAPO in isopropanol/water (80/20, *w*/*w*) at different temperatures for 1 h. (**d**) Degradation of mPEG_45_-CDPA under blue light irradiation (4.2 mW/cm^2^) as determined by UV–Vis spectroscopy (at 310 nm).

Figure 3a,b show polymerization kinetics of blue light-initiated alcoholic RAFT dispersion polymerization of mPEG_45_-PBzMA_100_ (20% *w*/*w*) at either 25 or 70 °C (465nm, 4.2 mW/cm^2^). It is found that the polymerization at 70 °C is significant faster than that at 25 °C. This is different from the case of alcoholic photo-PISA under 405 nm light irradiation [32]. To eliminate the influence of photo-initiator, the thermal stability of BAPO at 25 and 70 °C was checked. Figure 3c shows UV–Vis absorbance spectra of BAPO in isopropanol/water (80/20, *w*/*w*) at either 25 or 70 °C for 1 h. The UV–Vis absorbance spectra were almost identical, suggesting that BAPO is stable in isopropanol/water mixture at these temperatures. Therefore, we speculate the effect of temperature on the polymerization kinetics may be attributed to the photolysis of macro-RAFT agent under 465 nm light irradiation. The degradation degree of mPEG_45_-CDPA in isopropanol/water was then evaluated by monitoring the peak value at 310 nm under blue light irradiation (465 nm, 4.2 mW/cm^2^). As shown in Figure 3d, around 13% mPEG_45_-CDPA was degraded at 25 °C after 150 min of 465 nm light irradiation. When the temperature was increased to 70 °C, the degree of mPEG_45_-CDPA degradation was increased significantly to around 25% after 150 min of 465 nm light irradiation. It is well-known that the degradation of RAFT agent leads to the generation of free radicals [37]. Therefore, increasing the temperature will lead to faster blue light-initiated alcoholic RAFT dispersion polymerization.

The main advantage of PISA is that a variety of morphologies can be produced by changing reaction parameters, including monomer concentration, DP of the macro-CTA, and DP of the core-forming block. Figure S4 shows TEM images of mPEG_45_-PBzMA_n_ polymer nano-objects prepared by blue light-initiated alcoholic RAFT dispersion polymerization of BzMA at room temperature. Spheres were obtained at a monomer concentration of 10% *w*/*w*. Further increasing the monomer concentration to 20% *w*/*w* or 30% *w*/*w* led to the creation of pure vesicles even when the DP of PBzMA was as low as 48. In the synthesis of mPEG_45_-PBzMA, we were unable to obtain worm-like micelles due to the short length of mPEG_45_. We then attempted to use longer macro-CTA (mPEG_113_-CDPA) in blue light-initiated alcoholic RAFT dispersion polymerization. However, only spheres were obtained in all studied cases (data not shown). Although the use of mPEG-based macro-CTA is beneficial for the reproducible preparation of macro-CTA with the same DP, it is inconvenient to change the molecular weight of macro-CTA as desired since the types of commercially available PEG polymers are limited.

**Figure 4:**
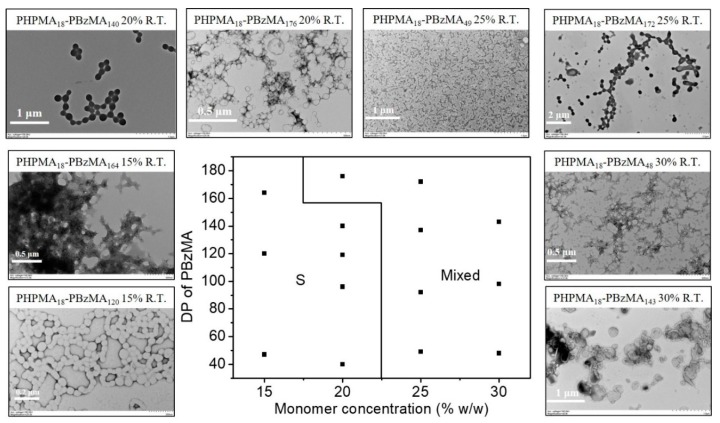
Representative TEM images and the morphological phase diagram for PHPMA_18_-PBzMA_n_ diblock copolymers prepared by blue light-initiated alcoholic RAFT dispersion polymerization of BzMA at 25 °C. Phase areas consist of spheres (S) and mixed morphology (Mixed).

**Figure 5:**
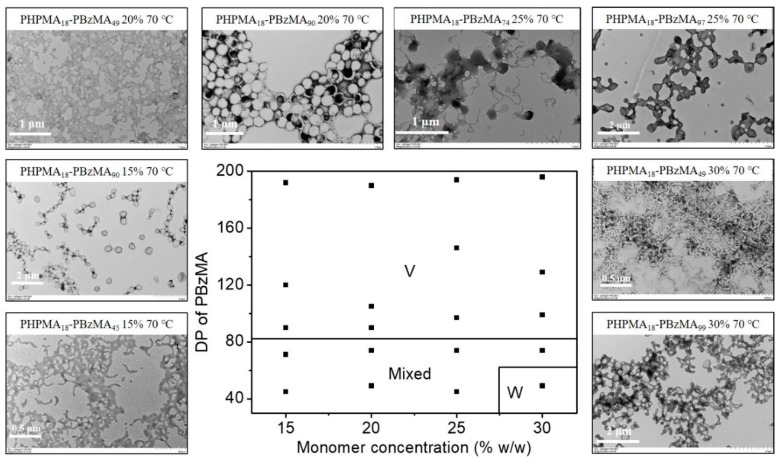
Representative TEM images and the morphological phase diagram for PHPMA_18_-PBzMA_n_ diblock copolymers prepared by blue light-initiated alcoholic RAFT dispersion polymerization of BzMA at 70 °C. Phase areas consist of worms (W), mixed morphology (Mixed) and vesicles (V).

To demonstrate the feasibility of the present blue light-initiated RAFT dispersion polymerization, polymethacrylate prepared by RAFT polymerization should also be employed as the macro-CTA since the length of macro-CTA can be controlled conveniently. PHPMA is a polymer that has good solubility in alcoholic solvents and can be used as macro-CTA of alcoholic RAFT dispersion polymerization [50]. PHPMA_18_-CDPA prepared by RAFT solution polymerization was then utilized to mediate the blue light-initiated RAFT dispersion polymerization of BzMA in an isopropanol/water (90/10, *w*/*w*) mixture. Two morphological phase diagrams of blue light-initiated alcoholic RAFT dispersion polymerization of BzMA at 25 and 70 °C were constructed by changing the DP of the PBzMA and the monomer concentration using PHPMA_18_-CDPA as the macro-RAFT agent. Mean DPs of PBzMA were determined by ^1^H NMR spectroscopy. When the reaction temperature was 25 °C (Figure 4), a large area of spheres was found in the phase diagram at low monomer concentrations (15% and 20% *w*/*w*). This can be explained by the reduced probability of fusion between spheres at low monomer concentrations, which has proven to be a key step for the formation of worms even vesicles. Further increasing the monomer concentration led to the formation of mixed morphologies. No pure worms or vesicles were formed at 25 °C in all studied cases. It should be noteworthy that it is possible to produce pure worms or vesicles at 25 °C by further varying reaction conditions, but this is not the objective in the present study. In contrast, the morphological phase diagram at 70 °C changed significantly in comparison to the one at 25 °C (Figure 5). In this case, a large area of the pure vesicles was found, and pure worms were formed at a DP of 49 (BzMA concentration of 30% *w*/*w*). Comparing two morphological phase diagrams, it is obvious that increasing the temperature promotes the formation of worms or vesicles in blue light-initiated alcoholic RAFT dispersion polymerization. The temperature-dependent morphological difference of the blue light-initiated alcoholic RAFT dispersion polymerization is because of two reasons. First, increasing the reaction temperature facilitates the fusion between polymer particles, which is critical for the creation of higher order morphologies. Second, the PBzMA chains have higher mobility at higher temperature, allowing the PBzMA chains to be swollen with BzMA easier, which contributes to the morphological evolution of spheres to higher order morphologies. Table S2 summarizes molecular weights and molecular weight distributions of the diblock copolymers. Similar molecular weights and molecular weight distributions were observed at 25 and 70 °C, which is consistent with the results as reported by Wenn and Junkers [51].

## 3. Conclusions

In conclusion, this work demonstrates a novel blue light-initiated alcoholic RAFT dispersion polymerization of BzMA using BAPO as the photo-initiator. Kinetic studies indicated that isopropanol/water mixture was a better solvent to achieve higher monomer conversions with higher rate of polymerization than that of ethanol/water or methanol/water. High monomer conversion (>95%) was achieved within 2 h of blue light irradiation (465 nm, 4.2 mW/cm^2^) in an isopropanol/water (80/20, *w*/*w*) mixture. THF GPC measurement confirmed that *M*_n_ increased linearly with monomer conversion. Moreover, low polydispersities (*M*_w_/*M*_n_ < 1.41) were observed during the polymerization, suggesting good control was maintained. Effect of light intensity on polymerization kinetics was also studied, and the rate of polymerization could be controlled easily by varying the light intensity.

Finally, the effect of temperature on the blue light-initiated alcoholic RAFT dispersion polymerization was investigated. Due to the faster degradation of mPEG_45_-CDPA at higher temperatures under blue light irradiation, increasing temperature led to a higher rate of polymerization in blue light-initiated alcoholic RAFT dispersion polymerization. Two morphological phase diagrams of blue light-initiated alcoholic RAFT dispersion polymerization of BzMA at 25 and 70 °C mediated by PHPMA_18_-CDPA were constructed. The results showed that increasing the temperature promoted the creation of higher order morphology.

## Supplementary Materials

The supplementary materials are available online at https://www.mdpi.com/2073-4360/11/8/1284/s1.
