# Supplementary material for: Blue Light-Initiated Alcoholic RAFT Dispersion Polymerization of Benzyl Methacrylate: A Detailed Study
Source: Polymers (Basel). 2019 Aug 1;11(8):1284. doi: 10.3390/polym11081284 (PMC6723682; doi:10.3390/polym11081284)
Supplement: Supplementary file 1 [file polymers-11-01284-s001.pdf]

Blue Light-Initiated Alcoholic RAFT Dispersion Polymerization of Benzyl Methacrylate: A Detailed Study

Dongdong Liu[a], Ruiming Zeng[a], Hao Sun[b], Li Zhang[a,c], Jianbo Tan[*a,c]

a. *Department of Polymeric Materials and Engineering, School of Materials and Energy, Guangdong University of Technology, Guangzhou 510006, China.*

b. *Department of Chemistry, Northwestern University, Evanston, IL, 60208, United States.*

c. *Guangdong Provincial Key Laboratory of Functional Soft Condensed Matter, Guangzhou 510006, China.*

**Corresponding author: tanjianbo@gdut.edu.cn*

Dongdong Liu and Ruiming Zeng contributed equally to this work.

EXPERIMENTAL SECTION

Materials. Benzyl methacrylate (BzMA, Aladdin), hydroxypropyl methacrylate (HPMA, Aladdin), monomethoxy poly(ethylene glycol) ($mPEG_{45}$, 2000 g/mol, Sigma-Aldrich), dicyclohexylcarbodiimide (DCC, Aladdin), 4-dimethylaminopyridine (DMAP, Aladdin), hydroquinone (Aladdin), sodium 2,2-dimethyl-2-silapentane-5-sulfonate (DSS, Aladdin), and phenylbis(2,4,6-trimethylbenzoyl)phosphine oxide (BAPO, Tianjin Jiuri) were used without further purification. 2, 2-Azobisisobutyronitrile (AIBN, Aladdin) was recrystallized from ethanol prior to storage under refrigeration at 4 °C. 4-Cyano-4-(dodecylsulfanylthiocarbonyl)sulfanylpentanoic acid (CDPA) was synthesized according to a published procedure.[1]

Synthesis of $mPEG_{45}$-CDPA. A solution of CDPA (8.07 g, 20 mmol) in 40 mL of anhydrous dichloromethane (DCM) was introduced in a dry flask under nitrogen atmosphere containing $mPEG_{45}$ (20.0 g, 10 mmol). Then a solution of DCC (4.12 g, 20 mmol) and DMAP (0.244 g, 2 mmol) in 10 mL of anhydrous dichloromethane was added dropwise to the reaction mixture at 0 °C. The esterification reaction proceeded under stirring at room temperature for 48 h. The polymer was collected by precipitation of the reaction mixture in cold diethyl ether, passing through a column, and finally dried at 45 °C under vacuum to obtain a yellow powder.

Synthesis of PHPMA macro-RAFT agent. HPMA (40.0 g, 277 mmol), AIBN (0.456 g, 2.77 mmol), and CDPA (5.60 g, 13.9 mmol) were added into a 250 mL round-bottom flask, and then dissolved with dioxane (60.0 g, 40% w/w) to form a homogeneous solution. 1,3,5-Trioxacyclohexane (2.5 g, 27.7 mmol) was added to the reaction mixture as an internal standard. The reaction mixture was then purged with nitrogen for 1 h, sealed, and then immersed in a 70 °C pre-heated oil bath for

120 min. The polymerization was quenched by immersion in ice-water and exposure to air. The polymer was precipitated by adding excess hexane (600 mL) and washed several times with additional hexane. The obtained product was then dried at 45 °C under vacuum overnight, and then analyzed by ^{1}H NMR spectroscopy and THF gel permeation chromatography (GPC). ^{1}H NMR measurement indicated a mean degree of polymerization of 18 for this macro-RAFT agent (denoted as $PHPMA_{18}$-CDPA), and THF GPC measurement confirmed its M_n = 4200 g mol^{-1} and M_w/M_n = 1.15.

Blue light-initiated alcoholic RAFT dispersion polymerization. In a typical experiment for the synthesis of $mPEG_{45}$-$PBzBA_{100}$ (20% w/w): BzMA (1.5 g, 8.5 mmol), $mPEG_{45}$-CDPA (0.20 g, 0.085 mmol), and BAPO (0.0119 g, 0.028 mmol) were weighed into a 25 mL round bottom flask. Then an isopropanol/water mixture (4.8 g/1.2 g, 80/20, w/w) was added into the flask to dissolve all reagents. The reaction mixture was purged with nitrogen for 20 min, and then irradiated by a visible light LED lamp (465 nm, 4.2 mW/cm^2) for 3 h. The reaction was quenched by exposure to air and the addition of a small amount of hydroquinone.

In a typical experiment for the synthesis of $PHPMA_{18}$-$PBzBA_{100}$ (20% w/w), BzMA (1.5 g, 8.5 mmol), $PHPMA_{18}$-CDPA (0.26 g, 0.085 mmol), and BAPO (0.0119 g, 0.028 mmol) were weighed into a 25 mL round bottom flask. Then an isopropanol/water mixture (5.4 g/0.6 g, 90/10, w/w) was added into the flask to dissolve all reagents. The reaction mixture was purged with nitrogen for 20 min, and then irradiated by a visible light LED lamp (465 nm, 4.2 mW/cm^2) for 3 h. The reaction was quenched by exposure to air and the addition of a small amount of hydroquinone.

Kinetic study of blue light-initiated alcoholic RAFT dispersion polymerization. BzMA (2.5 g, 14.2 mmol), $mPEG_{45}$-CDPA (0.34 g, 0.142 mmol), sodium 2,2-dimethyl-2-silapentane-5-sulfonate

(DSS,80 mg) and BAPO (0.0198 g, 0.047 mmol) were weighed into a 25 mL round bottom flask. Then an isopropanol/water mixture (8.0 g/2.0 g, 80/20, w/w) was added into the flask to dissolve all reagents. The reaction mixture was purged with nitrogen for 20 min, and then irradiated by a visible light LED lamp (465 nm, 4.2 mW/cm^2). Samples were withdrawn at predetermined time intervals by syringes under nitrogen, and the reaction was quenched by exposure to air and the addition of a small amount of hydroquinone. The samples were then analysis by ^{1}H NMR spectroscopy and gel permeation chromatography (GPC).

Characterization. The obtained dispersions were diluted 100-fold with isopropanol-water mixtures. A drop of the solution was placed on the copper grip for 1 min and then blotted with filter paper to remove excess solution. A drop of uranyl acetate solution (0.5 wt %) was soaked on the same copper grip for 1.5 min, and then blotted with filter paper to remove excess stain. Transmission electron microscope (TEM) observations were carried out on a Hitachi 7700 instrument operated at 120 kV.

The molecular weight and polydispersity of the block copolymers were measured by gel permeation chromatography (GPC) at 40 ^oC using a Waters 1515 GPC instrument with tetrahydrofuran (THF) as the mobile phase and Waters styragel HR1, HR4 columns. The flow rate of THF was 1 mL/min. Linear poly (methyl methacrylate) polymers with narrow molecular weight distributions were used as the standards to calibrate the apparatus.

^{1}H NMR spectra were recorded in $CDCl_3$ using a Bruker Avance III 400 MHz NMR spectrometer at a temperature of 25 ^oC. Note: The samples prepared by PISA were first diluted with methanol, and then dissolved in $CDCl_3$.

UV-vis spectra were recorded with a 1.0 cm quartz cuvette using a UV2450 spectrometer.

ADDITIONAL RESULTS

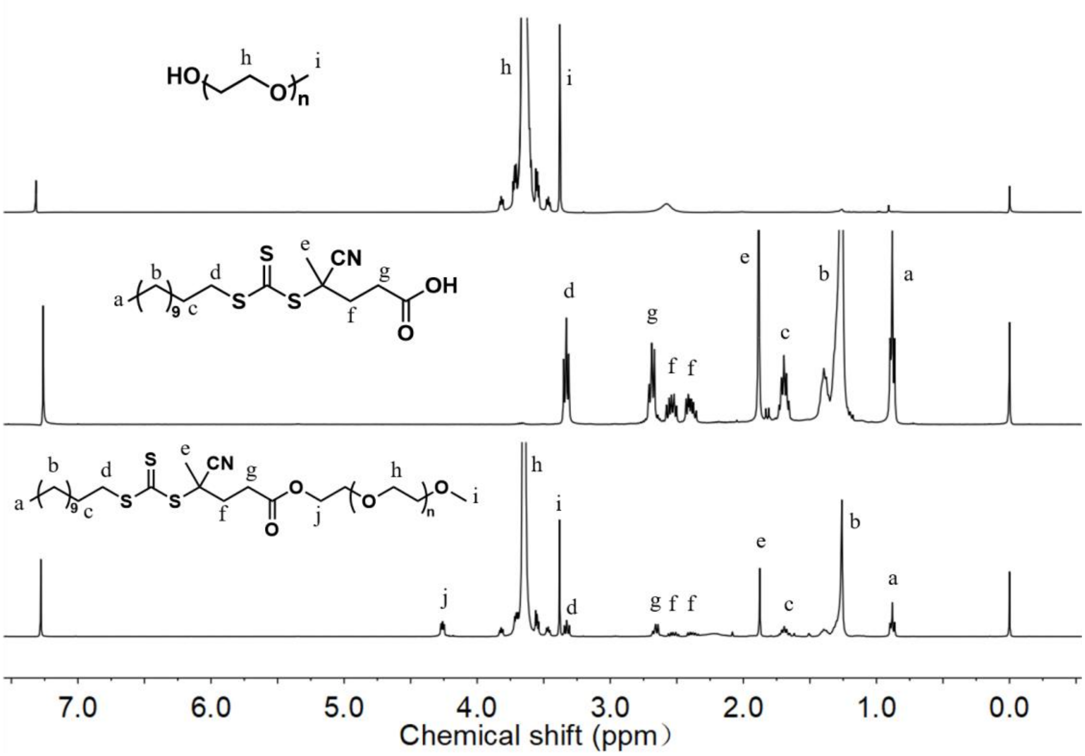

Figure S1. ^{1}H NMR spectra of mPEG$_{45}$, CDPA and mPEG$_{45}$-CDPA.

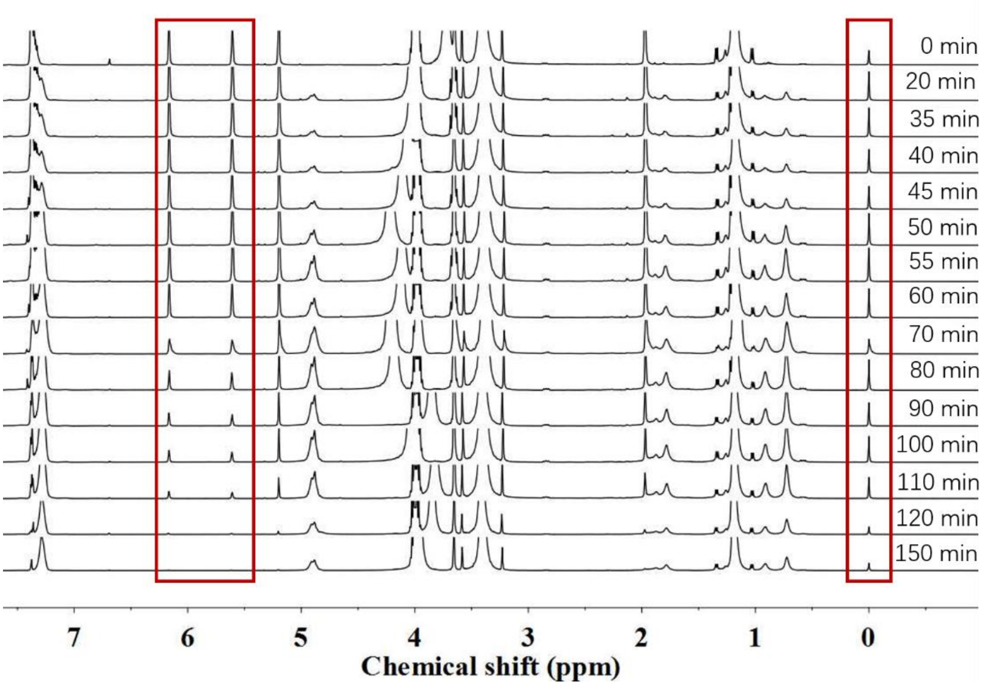

Figure S2. ^{1}H NMR spectra ($CDCl_3$) recorded during the blue light-initiated RAFT dispersion polymerization of BzMA using a mPEG$_{45}$-CDPA macro-CTA in an isopropanol/water (80/20, w/w) mixture (BzMA concentration of 20% w/w, target DP of 100). Sodium 2,2-dimethyl-2-silapentane-5-sulfonate (DSS) was used as the internal ^{1}H NMR standard.

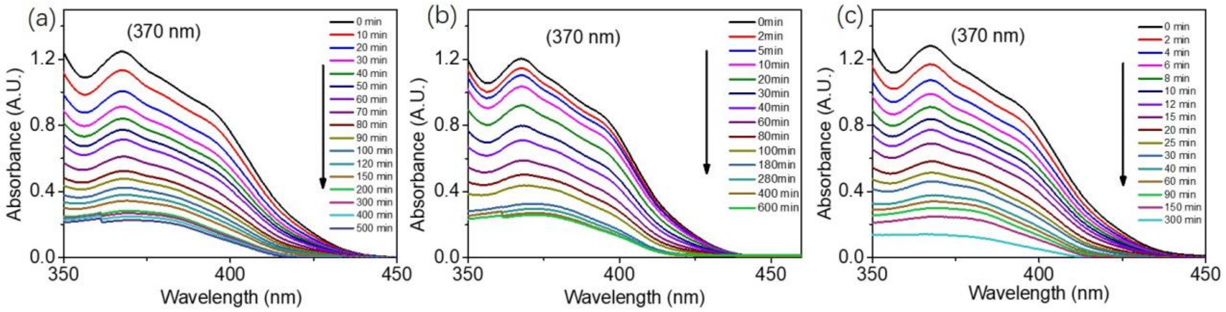

Figure S3. UV-vis absorbance spectra recorded for the decomposition of BAPO in isopropanol/water (80/20, w/w) under blue light irradiation (λ_{max} = 465 nm) with different light intensities. (a) 2.0 mW/cm^2; (b) 4.2 mW/cm^2; (c) 8.0 mW/cm^2.

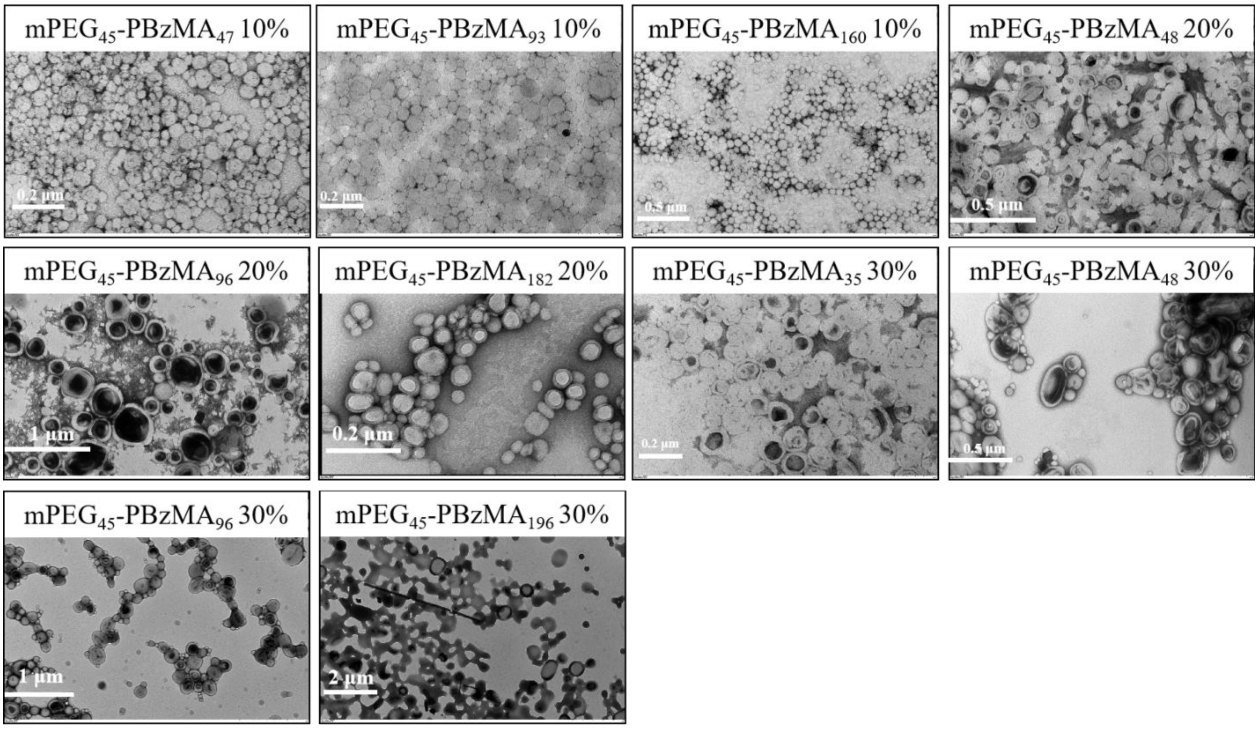

Figure S4. TEM images of mPEG$_{45}$-PBzMA$_n$ diblock copolymer nano-objects prepared via alcoholic blue light-initiated RAFT dispersion polymerization of BzMA in isopropanol/water (80/20, w/w) at 25 °C.

Table S1. Summary of GPC data obtained for a series of PHPMA$_{18}$-PBzMA$_{100}$ diblock copolymers prepared by blue light-initiated RAFT dispersion polymerization of BzMA in an isopropanol/water (90/10, w/w) mixture at a BzMA concentration of 25% w/w under different light intensities.[a]

Light intensity	Target composition	BzMA conversion	GPC	
			M_n (g mol^{-1})	M_w/M_n
2.0 mW/cm^2	mPEG$_{45}$-PBzMA$_{100}$	93	13275	1.24
4.2 mW/cm^2	mPEG$_{45}$-PBzMA$_{100}$	95	13520	1.28
8.0 mW/cm^2	mPEG$_{45}$-PBzMA$_{100}$	88	12758	1.28

[a] All polymerizations were conducted using a macro-CTA/photoinitiator molar ratio of 3.0. Monomer conversions were measured using ^{1}H NMR spectroscopy, and GPC data were recorded via THF GPC.

Table S2. Summary of reaction temperature, synthesis conditions, monomer conversions, GPC data, and TEM characterization obtained for a series of $PHPMA_{18}$-$PBzMA_n$ diblock copolymers prepared by blue light-initiated RAFT dispersion polymerization (light intensity of 4.2 mW/cm^2) of BzMA in an isopropanol/water (90/10, w/w) mixture at a BzMA concentration of 25% w/w.[a]

Reaction temperature (℃)	Target composition	BzMA conversion (%)	Actual PBzMA DP	GPC		TEM
				M_n (kg/mol)	M_w/M_n	morphology
	$PHPMA_{18}$-CDPA	-	-	4.2	1.15	
25	$PHPMA_{18}$-$PBzMA_{50}$	98	49	11.2	1.25	spheres + worms
25	$PHPMA_{18}$-$PBzMA_{100}$	92	92	15.9	1.48	spheres + worms + vesicles
25	$PHPMA_{18}$-$PBzMA_{150}$	91	137	23.0	1.65	spheres + vesicles
25	$PHPMA_{18}$-$PBzMA_{200}$	86	172	38.5	1.65	spheres + vesicles
70	$PHPMA_{18}$-$PBzMA_{50}$	98	49	10.2	1.41	Spheres + worms
70	$PHPMA_{18}$-$PBzMA_{100}$	97	97	17.1	1.42	vesicles
70	$PHPMA_{18}$-$PBzMA_{150}$	97	146	20.3	1.60	vesicles
70	$PHPMA_{18}$-$PBzMA_{200}$	97	194	25.4	1.64	vesicles

[a] All polymerizations were conducted using a macro-CTA/photoinitiator molar ratio of 3.0. Monomer conversions were measured using ^1H NMR spectroscopy, and GPC data were recorded via THF GPC.

REFERENCES

(1) Moad, G.; Chong, Y. K.; Postma, A.; Rizzardo, E.; Thang, S. H. *Polymer* **2005**, *46* (19), 8458–8468.
